# Have we lost sleep? A reconsideration of segmented sleep in early modern England

**DOI:** 10.1017/mdh.2023.14

**Published:** 2023-04

**Authors:** Niall Boyce

**Affiliations:** Department of English, Theatre and Creative Writing, School of Arts Birkbeck, University of London, 43 Gordon Square, London WC1H 0PD, UK

**Keywords:** Early modern, sleep, night, first sleep, segmented sleep, insomnia, nocturnal

## Abstract

The theory that the people of the early modern period slept in well-defined segments of ‘first’ and ‘second’ sleeps has been highly influential in both scholarly literature and mainstream media over the past twenty years. Based on a combination of scientific, anthropological and textual evidence, the segmented sleep theory has been used to illuminate discussions regarding important aspects of early modern nocturnal culture; mainstream media reports, meanwhile, have proposed segmented sleep as a potentially ‘natural’ and healthier alternative to consolidated blocks of sleep. In this article, I re-examine the scientific, anthropological and early modern literary sources behind the segmented sleep theory and ask if the evidence might support other models of early modern sleep that are not characterised by segmentation, while acknowledging that construction of such models is by nature limited and uncertain. I propose a more diverse range of interpretations of early modern texts related to sleep, with important implications for medical and social history and literary scholarship.

## Introduction

Roger Ekirch’s theory that the people of the pre-industrial age split their sleep into two distinct periods, filling gaps of up to an hour inbetween with social activity or private meditation, has been widely accepted by historians, literary scholars and the general public in the two decades following its original publication. The segmented sleep theory proposes that:

Until the modern era, up to an hour or more of quiet wakefulness midway through the night interrupted the rest of most Western Europeans […] Families rose from their beds to urinate, smoke tobacco, and even visit close neighbors. Remaining abed, many persons also made love, prayed, and, most important, reflected on the dreams that typically preceded waking from their ‘first sleep’. Not only were these visions unusually vivid, but their images would have intruded far less on conscious thought had sleepers not stirred until dawn. […] In addition to suggesting that consolidated sleep, such as we today experience, is unnatural, segmented slumber afforded the unconscious an expanded avenue to the waking world that has remained closed for most of the Industrial Age.^1^

This model of pre-modern ‘first’ and ‘second’ sleep has caught the attention and imagination of academic and popular authors alike. For example, historian Craig Koslofsky writes that ‘the experience of segmented sleep seems to have been familiar to all medieval and early modern Europeans’ and that the wakeful interval between sleeps was ‘a demarcated period of nocturnal activity in the middle of long nights’.^2^ In his book on *Dreams, Sleep, and Shakespeare’s Genres*, literary scholar Claude Fretz uses Ekirch’s evidence to make the case that a gap between sleeps allowed for review and contemplation of dreams, meaning that early modern dreams were ‘more powerful phenomena than our modern experience of dreaming might lead us to believe’.^3^ Historians Tara Hamling and Catherine Richardson state that ‘Both phases of sleep lasted roughly the same amount of time, individuals waking some time around midnight […]. Thus, some authors assumed a natural waking at this time and attempted to guide thought to spiritual ends.’^4^

The evocative images conjured in Ekirch’s prose – of ‘Families’ who ‘rose from their beds to urinate, smoke tobacco, and even visit close neighbors’ – have also become commonplace in material aimed at a general audience, possibly going beyond Ekirch’s original interpretation and intentions. The Folger Shakespeare Library’s family worksheet on sleep, produced to accompany a 2009 exhibition, asks its readers:

Can you imagine waking up at midnight, hanging out with your friends for an hour, and then going back to sleep again? For Elizabethans, night-time slumber was divided into ‘first’ and ‘second’ sleeps, separated by a time when people would wake up and take part in activities such as socializing, reading, or praying.^5^

In their popular market-oriented history book *What We Did in Bed: A Horizontal History* (2019), Brian Fagan and Nadia Durrani speculate that ‘our modern desire to deny this “natural” [segmented] sleep rhythm’ might have ‘led to our current multibillion-dollar reliance on sleeping pills’, and ask, ‘Could we solve our sleep problems simply by understanding how we used to do things?’^6^ A BBC website item in 2012 asserted that ‘the majority of doctors still fail to acknowledge that a consolidated eight-hour sleep may be unnatural’ and concluded that ‘the next time you wake up in the middle of the night, think of your pre-industrial ancestors and relax. Lying awake could be good for you.’^7^ In 2022, an article in *The Sunday Times* stated that ‘Before the Industrial Revolution, segmented sleep was accepted as normal. Insomniac cavemen would stay awake on watch for danger and from the Middle Ages to medieval times [*sic*] people would sleep in two shifts’; it went on to claim that ‘more people are turning to a segmented sleeping schedule, which typically involves two four-hour stages of sleep, with a break of a few hours in the middle’.^8^ Robert Harris’s 2019 novel about a future society that has reverted to a medieval way of life is entitled *The Second Sleep*, quotes Ekirch in its epigraph, and includes a scene in which the visiting protagonist wakes between first and second sleeps to find the locals socialising: ‘In Exeter, the custom between the first and second sleeps is to stick to our rooms. Yet the villagers here are abroad.’^9^

Perhaps the segmented sleep theory appeals because it presents a radical historical alternative to a pattern of consolidated sleep that is so accepted in the modern-day Western world as to seem a completely natural biological process. Certainly, segmented sleep appears to be firmly established as part of the modern-day cultural understanding of the pre-industrial world. The theory that human sleep is naturally segmented has even, as the news reports quoted above indicate, been used by some as health advice. In this context, it is helpful to return to Ekirch’s original research and to search for and flag up any ambiguities that might alter our readings of the texts he cites in support of his argument.

Ekirch’s research has undoubtedly been of great value to social, cultural and literary historians, both in emphasising the importance of sleeping practices in interpreting historical cultures and in calling into question basic assumptions about such practices. The purpose of the present article is not to altogether deny the existence of segmented sleep in the past, and specifically the early modern period. It is, instead, to demonstrate that some of the scientific evidence, anthropological evidence and in particular the early modern texts cited in Ekirch’s 2001 article that apparently refer to segmented sleep might be interpreted in a different manner. An apparent mountain of evidence for segmented sleep which has been carefully gathered and curated by Ekirch might also provide rich details regarding a diversity of sleeping patterns and practices in the early modern period. The latter part of this article re-evaluates some of the copious material from the 2001 article ‘Sleep We Have Lost’. I have focused in particular on material from England in the sixteenth to the eighteenth centuries, first as this makes up a large amount of the material used by Ekirch to support his argument, and second, as this provides a comparatively coherent set of texts to analyse, in terms of linguistic, historical and geographic boundaries, while also being a rich source of pre-industrial writing.^10^ Ekirch himself has noted that ‘Allusions to “first” and “second sleep” are plentiful in early modern texts’.^11^

‘Sleep We Have Lost’ was by no means Ekirch’s final word on the subject of segmented sleep. In subsequent articles he has made modifications to his argument and provided additional evidence. However, this article focuses on the 2001 publication because it is arguably his most influential articulation of the theory, and the essence of his approach – citing scientific and anthropological texts, and quoting from a vast number of pre-industrial (and selected post-industrial) texts which apparently refer to segmented sleep – has remained unchanged.^12^ For example, Ekirch’s 2015 *Past and Present* paper ‘The Modernization of Western Sleep’, like ‘Sleep We Have Lost’, uses the retrieval and analysis of numerous references to ‘first’ and ‘second’ sleeps from mainly printed texts to explore historical sleeping patterns.^13^

I am not the first to strike a note of caution, nor to suggest that the early modern night might have seen a wide diversity of sleeping practices and patterns. For example, Janine Rivière notes that ‘although A. Roger Ekirch suggests premodern people typically experienced a pattern of segmented sleep, this is not discussed in manuals of health or discussions of the proper regimens of sleep.’^14^ It is certainly striking that while Ekirch argues that his evidence consists of ‘fragments’ and ‘shards’ in ‘sources ranging from depositions and diaries to imaginative literature’, he must ‘piece together’ to construct a pattern of biphasic, segmented early modern sleep, Riviere’s research has identified no single, unambiguous early modern medical discussion of segmented sleep.^15^ Furthermore, although Sasha Handley reads some textual evidence as congruent with segmented sleep, she also cites the German-language work of Gabriele Klug when expressing doubt as to ‘whether the linguistic meanings of “first” and “second” sleep do in fact correspond to a pattern of segmented slumber.’^16^ In private correspondence, Klug (now Schichta) has informed me that:

I was able to find some matching references in the Middle High German texts I analyzed: There are several records of a ‘first sleep’ (though none of a ‘second’ sleep). However, I used to interpret these passages with regard to the ‘common knowledge’ (which, by the way, still seems to be an issue in our days) that sleep before midnight is deeper and ‘better’ than sleep after midnight. In medieval German literature I found no references to actual periods of wakefulness that would have separated first and second sleep (apart from examples related to religious practice, i.e. if a person rises to celebrate the Liturgy of the Hour).^17^

Research such as Schichta’s does not invalidate the concept of routinely divided first and second sleeps in early modern England; it does, however, raise the question as to why such practices left little, or equivocal, evidence in German texts. Handley proposes that ‘Much evidence does exist to support the wide-spread practice of biphasic sleep but such routines were not uniformly characteristic of early modern habits’.^18^ The present article goes further still, arguing that this supposedly supportive evidence might itself be re-interpreted in a way that reveals ambiguities and contradictions, pointing to a greater complexity than a simple and widespread pattern of ‘first’ and ‘second’ periods of sleep. It is possible that sleep did occur in two phases in some cases in early modern England; but reading early modern texts with the assumption that segmented sleep was a routine and widespread practice might lead to misinterpretation or, rather, restricted interpretation of the evidence. For the avoidance of doubt, this is not to argue that Ekirch arrived at his views on segmented sleep before his analysis of the copious evidence he provides. But it is to say that carefully re-examining the texts Ekirch presents might reveal new interpretations that do not rely on the segmented sleep model. Segmented sleep may be just one framework, rather than the sole framework, for understanding texts concerning pre-industrial sleep.

Indeed, Ekirch’s summary of his own thesis—cited at length above—is not without ambiguity. The phrase ‘up to an hour or more’ is unclear: the wording could mean ‘around an hour’ but might also encompass anything from a few seconds of wakefulness to an extended period of activity. The implication seems to be that a gap of an hour or so between phases of sleep was normal practice for the majority of people (however, the activities he mentioned require very different amounts of time and wakefulness: from urinating, to making love, to visiting neighbours).^19^ More creative interpretations of Ekirch’s work, such as the Robert Harris novel cited above, portray nocturnal waking and activity as a collective phenomenon (possibly taking their cue from the reference to ‘Families’); however, this is not a conclusion explicitly drawn by Ekirch himself.

Ekirch’s assertion that dreams in the first phase of sleep were ‘unusually vivid’ and more likely to be remembered because sleep was broken is similarly questionable. It is unclear whether Ekirch means that dreams during the first sleep were richer experiences than dreams in the second sleep, whether there was something about the gap between first and second sleeps that meant memorisation of first-sleep dreams was more likely than that of second-sleep dreams, or both.

Evidence from both contemporary sleep research and early modern texts qualifies somewhat the proposition that first-sleep dreams might have had ‘unusually vivid’ qualities.^20^ Modern-day sleep studies indicate that human beings usually cycle in and out of REM (rapid eye movement) sleep throughout the night, with a preponderance of REM in the latter part of the night; REM is the sleep phase associated with vivid narrative dreams.^21^ Although there might be difficulties in the wholesale application of neuroscientific findings in modern-day humans to the brains and bodies of early modern individuals, this evidence suggests that there is a human tendency for more vivid dreams to be experienced towards the hours of morning, rather than in the initial period of sleep. Furthermore, this evidence is consistent with the common early modern belief that morning dreams held the most reliable truth value, occurring, as they did, at a point when, as Rivière puts it, ‘the body had completed digestion, allowing the soul to commune more freely with spirits’.^22^ The term ‘morning’ is, of course, inexact, although the sources cited by Rivière appear to refer to the period leading up to the dawn. Even the physician Richard Haydocke (1569 or 1670, *c.*1642), who rejected the idea of morning dreams as prophetic, acknowledged their characteristic clarity:

ye morninge dreames begotten of ye milde vapours of ye second concoction, are most sincere & pure: insoemuch yt some haue esteemed them ye truer; whereof there is noe great reason. They may bee the more intire and aptly composed, as of poeticall fictions some may be more artificially handled then others, and yet none of them true.^23^

The phrase ‘intire and aptly composed’ might be suggestive of dreams that have some form of memorable—albeit bizarre—narrative coherence. Incidentally, Haydocke’s use of the phrase ‘second concoction’ suggests another ambiguity that problematises understanding of first and second sleeps, and is discussed in further detail below; it is unclear whether the existence of two or more biological phases in the early modern conception of sleep necessarily implied that there would be a significant gap of regained consciousness between these phases, or whether the phrases ‘first’ and ‘second’ might be more akin to the descriptors ‘early’ and ‘late’.

Ekirch’s argument that dream ‘images would have intruded far less on conscious thought had sleepers not stirred until dawn’ is plausible; it is impossible to consciously reflect on mental experiences while one is still in a state of unconsciousness (putting aside cases of lucid dreaming). However, it is unclear that a midnight phase of wakefulness would have led to the greater ability of the individual to memorise the details of dreams. Ekirch proposes that a period of wakefulness in the middle of the night would have allowed for the consolidation of dreams in long-term memory: ‘Had pre-industrial families not stirred until dawn, remaining instead asleep in their beds, many of these visions of self-revelation, solace, and spirituality would have perished by the bedside—some lost in the throes of sleep, others dissipated by the distractions of a new day’.^24^ With a little preparation, it is also possible that artificial light and writing materials close to hand might have aided the memorisation of dreams. However, the new day would also have brought with it the means to rapidly record and reflect upon dreams, with even easier access to light and writing materials; indeed, the notebooks of astrological physician Simon Forman include references to morning dreams such as that dated 4 January, 1594: ‘in the morninge lyinge in my bed I dremt howe I was in a place wher too men were readinge a bocke of the philosophers stone’.^25^ Furthermore, the evidence that morning dreams were held to be potentially significant experiences, or at least notably rich and coherent, suggests that the ‘distractions of the new day’ did not necessarily mean that dreams before dawn faded rapidly into oblivion and insignificance. Of course, it is impossible to quantify the factors that argue for midnight dreams being more memorable (a waking period of reflection) versus morning dreams being more memorable (REM sleep patterns, cultural significance, availability of light and writing materials, possible ability to relate the dream to others). The point, however, is that if we are to ask why dreams formed an important part of early modern culture, it is important to look beyond segmented sleep as an explanation. This is not to suggest that Ekirch himself proposes that segmented sleep is the sole reason for the manner in which early modern culture treated dreams; however, as the work cited at the beginning of this article suggests, it is an explanation to which early modern scholarship readily resorts.^26^

Finally, Ekirch’s suggestion that ‘consolidated sleep, such as we today experience, is unnatural’, while an eye-catching assertion, is ambiguous in both its precise meaning and implication. It is unclear where the line of ‘natural’ versus ‘unnatural’ is drawn in a species which has such a diverse range of technologies and social practices. It is similarly questionable – to address the arguments made in the popular histories and news reports cited above – whether a ‘natural’ sociobiological practice is necessarily better for one’s health than an ‘unnatural’ one.^27^ For example, pasteurisation, antibiotics, anaesthetics and vaccination are ‘unnatural’ in that they are the inventions of a complex, technology-rich society; few would argue, however, that their absence made the people of the early modern period healthier. Rather than pit the ‘natural’ against the ‘unnatural’, it is possible to acknowledge that sleeping practices have varied and continue to vary across societies, and to study the ways in which these variations interact with religious, scientific and cultural frameworks. This article re-assesses the original evidence for segmented sleep proposed by Ekirch with the intention not of ruling out the possibility of segmented sleep, but of determining whether alternative readings of this evidence—and hence, alternative accounts of early modern sleep—are possible.

## The science of segmented sleep

The scientific and anthropological evidence that Ekirch employs to support the idea of segmented sleep is best described as selective. The single scientific study cited is a 1993 paper by Thomas Wehr, via its report in the *New York Times.* Ekirch writes that Wehr’s study demonstrated that ‘human subjects, deprived at night of artificial light over a span of several weeks, eventually exhibited a pattern of broken slumber—astonishingly, one practically identical to that of pre-industrial households.’^28^ However, this finding is questionable.

The scientific paper on which the *New York Times* report appears to be based is a relatively small study of 16 participants, aged between 20 and 36 years; 15 were male, and 1 female.^29^ The study shifted these participants from a ‘long-day photoperiod regime (16 h light/activity, 8 h dark/rest) for 1 wk’ to ‘a short-day regime (10 h light/activity, 14 h dark/rest) for 4 wk’ over a 2-week interval. During the dark period, participants were ‘confined alone in a windowless dark room’ and ‘instructed to remain at bed rest and to sleep whenever possible […] except when it was necessary to use an adjoining dark bathroom.’ Wehr and colleagues also note that ‘Activities, such as exercising or listening to music, were not permitted during the dark period.’ During the ‘daily light period’, participants ‘went about their normal activities exposed to the ambient artificial and natural light in their normal environment.’^30^ The extent to which this experimental regime can be considered a simulation of sleeping conditions in the pre-industrial—and specifically the early modern—world is dubious; the *New York Times* report states that the lighting pattern was used ‘to recapitulate prehistoric sleep conditions’ on ‘a schedule that approximates what prehistoric people in the middle latitudes would have experienced in the dead of winter.’^31^ The strictly enforced ban on any form of light or activity at night in a ‘windowless dark room’ has clear differences from the experience of a few hours of evening wakefulness with the light from candles or a fire, followed by sleep in one’s own bed. Furthermore, given the seasonal changes in the hours of daylight, no one in early modern England would have experienced 14 hours of darkness as a regular fixture all year round. While it was clearly not Wehr’s intention to produce conditions that mimicked the early modern night—and nor does Ekirch propose this is the case—one has to question what the significance of a finding of biphasic sleep under these conditions, even if confirmed, would be. It is unclear to what extent an inherent pattern of biphasic sleep, even emergent under light deprivation, would persist under the influence of specific cultural sleeping practices such as the material conditions of sleep (beds and bedding, solitary or communal), diet, medicines and prayer and meditation.

Whatever the case, the evidence regarding a natural pattern of split sleep is unclear; although the paper states that sleep was ‘typically separated into two fragments with an interval of wakefulness between them’, the relevant figure does not demonstrate this conclusively, and indeed its legend states that ‘In long nights, sleep generally separated into ≥2 [ie. two or more] fragments and often exhibited a symmetrical bimodal pattern of distribution’. The ‘bimodal pattern’, in other words, is subjectively assessed and neither clearly defined nor quantified.^32^ Incidentally, a 2013 study by Kenneth Wright and colleagues, in which eight participants (two females) were studied under 1 week of normal daily routines with exposure to electrical lighting followed by 2 weeks of camping in summer (a 14 hour 40 minute light: 9 hour 20 minute dark cycle with only exposure to firelight at night), found that ‘natural’ lighting conditions affected the timing of the circadian clock; but biphasic sleep was not reported.^33^ However, small studies of relatively short duration should not of themselves lead one to dismiss Wehr’s data. To fully resolve this issue, larger-scale studies and possibly systematic review of the scientific evidence are needed.

As with the data cited from Wehr’s work, the anthropological evidence that Ekirch presents is of questionable relevance to sleep in the early modern world. He writes that ‘Anthropologists have found villages of the Tiv, Chagga, and G/wi, for example, in Africa to be surprisingly alive after midnight with newly roused adults and children.’ He cites ‘a study in 1969’ of the Tiv in Nigeria that ‘recorded, “At night, they wake when they will and talk with anyone else awake in the hut”’, and adds that ‘The Tiv even employ the terms “first sleep” and “second sleep” as traditional intervals of time.’^34^ Leaving aside the issue as to whether a twentieth-century Nigerian society has sufficient features in common with early modern European culture to allow one to throw light upon the other, the text cited by Ekirch is again open to more than one interpretation.

While Paul Bohannan’s 1953 study documents the use of the terms ‘first sleep’ and ‘second sleep’ by the Tiv, it is not clear that these terms are used to describe the sort of sleeping practice Ekirch proposes, that is, two neatly differentiated segments of sleep with a break in the middle:

Tiv are much less specific about time during the night. The time between dusk and about 10 o’clock is called ‘sitting together’ (*teman imôngo*). After that follows ‘the middle of the night’ (*helatô tugh*), which overlaps with the ‘time of the first sleep’ *(icin i mnya môm*); ‘the time of the second sleep’ (*acin a mnya ahar*) is about 3 AM or a bit later. The pre-dawn breeze (*kiishi*) gives its name to the period just before dawn.^35^

This paragraph is part of a study of concepts of time among the Tiv. It immediately follows a section concerning the precision with which the Tiv divide up the daylight hours through the position of the sun; the point is that the hours of darkness are less well defined, not that they are divided into clear and distinct periods. The article is not concerned with sleeping practices as such, and thus it is not clear that Bohannan is indicating that there are two periods of sleep with a gap of wakefulness, or whether the ‘first’ and ‘second sleep’ might simply be used in a way that is broadly synonymous with earlier and later phases of the night.

Meanwhile, the quotation from the ‘study in 1969’ that states ‘At night they wake when they will and talk with anyone else awake in the hut’ indicates simply that some individuals do not sleep through the night, and that the sleeping arrangements of the Tiv are such that it is possible they might find another person awake. This might indicate occasionally broken sleep for a certain number of individuals rather than widespread observance of clear periods of segmented sleep. The anthropological evidence is, as with the scientific evidence presented, neither clearly generalisable nor unequivocal in its documentation of segmented sleep.

## Re-reading the texts: the meaning of ‘first’ and ‘second’ sleep

The bulk of the evidence Ekirch presents is not scientific or anthropological, but consists of excerpts from a variety of early modern texts. The picture constructed is based on, as noted above, a proliferation of what he calls ‘shards’ fitted together, rather than detailed analysis of any single text. While this method has the strength of gathering material from a wide range of sources, it has an important limitation: the various quotations that Ekirch presents in his study might support the idea of segmented sleep if read with that concept in mind, but may also suggest other possibilities, such as idiosyncratically broken sleep, or specific circumstances in which light and interrupted sleep was to be expected.

Poor-quality sleep was not uncommon in early modern England. Rivière notes the data gleaned by Michael MacDonald from the medical notebooks of Richard Napier that reveal 20 percent of all cases involved trouble sleeping; 2.7 percent of these cases included a complaint of ‘fearful dreams’, suggesting that at least some of these individuals were troubled by undesired waking (referred to today as middle and terminal insomnia) rather than, or in addition to, the inability to fall asleep (initial insomnia).^36^ Indeed, some material cited by historians in support of routinely segmented early modern sleep might also be read simply as evidence of undesirable broken sleep. For example, Hamling and Richardson cite a prayer from Thomas Bentley’s *The Monument of Matrons* (1582) to illustrate their point that ‘some authors assumed a natural waking’ in the middle of the night.^37^ The poem in question is entitled ‘At Mid-night, If You Happen to Awake’, and the author attributes nocturnal waking to divine action: ‘Euen now in the night season, while thou holdest mine eies waking, I saie, will I thinke of thee my creator’.^38^ The phrase ‘if you happen to awake’ suggests preparation for a possible eventuality, rather than for an inevitable part of the night’s schedule. Moreover, ‘Euen now’ suggests that this nocturnal waking is troublesome and undesirable, rather than routine; Bentley is praising God despite holding Him responsible for his inability to return to sleep.

Another example that illustrates the importance of context is Ekirch’s citation of Montague Summers’s English translation of Noel Taillepied’s *A Treatise of Ghosts*, which describes the period ‘about midnight when a man wakes from his first sleep’.^39^ The full sentence reads: ‘In all ages throughout history has it been recorded that disembodied Spirits have appeared, as well by day as night, but more often about midnight when a man wakes from his first sleep and the senses are alert, having taken some repose’. The passage follows a description of how ‘the clear unclouded vision of a child often perceives spiritual visitants whom older eyes cannot discern’ and hence carries the implication that the beneficial first phase of sleep makes the perception of ghosts more likely.^40^ The reference might mean that ghosts tend to show up around midnight, when people normally wake from the first period of segmented sleep; another interpretation is that the individual has incidentally woken up from a deep and restful first phase of sleep and is thus able to see the ghost; another interpretation might be that the awakening is related to the arrival of the ghost. Elsewhere in Taillepied’s text, there is an allusion to being woken from sleep by a haunting; in a section on how spirits may haunt those responsible for their deaths, he quotes Virgil’s version of Dido’s promise to Aeneas that she will ‘disturb thy Sleep’ as an ‘angry Ghost’.^41^ It is clear that Taillepied is claiming the first phase of sleep is particularly restful and beneficial; it is less clear that this is unambiguous evidence for segmented sleep.

To take a further example, in this case one where detailed biographical information about the subject is available, Ekirch’s collection of segmented sleep references online includes an excerpt from a letter from Erasmus to Johann Choler, dated August 1535: ‘I accomplished this work, without being able to give it all the care it should have taken, but [during] hours of the afternoon, walking, while my families ate, sometimes in bed, while waiting for the second sleep.’^42^ Ekirch does not, however, note that at this point in Erasmus’ life, he was experiencing health problems, notably chronic pain caused by gout, which interfered with his sleep.^43^ It is therefore uncertain that an excerpt from Erasmus’ work at this stage in his life can be taken as representative of a normal or expected sleeping pattern; the point he is making, that his work has occurred in snatched time rather than with sufficient care and focus, suggests that wakefulness and activity between first and second periods of sleep is not necessarily a social or personal norm.

Evidence that waking from a first sleep into a period of alertness might have been associated with some distress is provided by Sasha Handley’s citation from prayer books, including a 1749 edition of Bishop of Bangor Lewis Bayly’s *New Practice of Piety*, which, in Handley’s words, ‘included set prayers for distinct phases of the night: for undressing, lying down in bed, settling to prayer, waking between the first and second sleep, and for accidental waking, which might be due, for example, to the noise of a striking clock.’^44^ However, the relevant page of the *New Practice* does not use the language of a ‘first’ or ‘second’ sleep, but refers to a prayer ‘*To be used* in the Night *when you* awake, *or cannot sleep*’, which begins ‘Stand in awe and sin not: commune with your own heart upon your bed, and be still.’^45^ It is not clear from the text whether or not this waking is a routine part of a segmented pattern of sleep, or a common, if unwanted, nocturnal experience (as the phrase ‘*cannot sleep*’ would suggest a term that covers both initial difficulties falling asleep and sleeping difficulties following nocturnal waking). While ‘when you awake’ and ‘cannot sleep’ are distinguished from one another, their grouping together within this text suggests that both might be undesirable or even distressing states. This impression is reinforced by the fact that this prayer and the prayer about the striking clock are gathered under the heading ‘Ejaculations’, which at the time of publication denoted ‘The putting up of short earnest prayers in moments of emergency’ and ‘A short prayer “darted up to God” […] in an emergency.’^46^

Moreover, nocturnal waking was not necessarily a feature unique to or uniquely bothersome in early modern England. A 2010 survey recorded that 31.2% of the general European population reported waking up at least three nights per week, and that 7.7% had difficulty resuming sleep. Nocturnal awakenings, in other words, are highly prevalent in present-day Europeans.^47^ The specific drivers behind poor and broken sleep have changed over the past 500 years; however, it is not necessarily the case that there was a time of ‘natural’ segmented sleep, in which a clear gap between two sleeps was an anticipated part of one’s nightly routine, that has now been lost.

Detailed analysis of a sample of Ekirch’s sources reveals alternative readings; while segmented sleep cannot be ruled out, neither is it the sole possible explanation for the terminology or circumstances described within the texts. Ekirch states that the first segment of sleep was referred to as a ‘first sleep’ (or, less frequently, ‘first nap’ or ‘dead sleep’).^48^ However, as noted above (and discussed in detail below), this term might simply describe the first phase of a continuous night’s sleep; even if a period of wakefulness is implied, it is not always clear that this was a regular part, ‘up to an hour’, of the normal early modern night. Indeed, some sources cited by Ekirch describe distinctly unusual circumstances. For example, Ekirch quotes William Davenant’s *The Unfortunate Lovers*, in which Rampino (‘A young gallant souldier, much indebted and vexed by Creditors’) states he is ‘More watchfull than/A sicke Constable after his first sleepe/On a cold bench’.^49^ The figure of speech here is unclear. It might be drawing a parallel between the officer sleeping on duty and the usual domestic pattern of segmented sleep. However, the implication could also be that, after dropping off during the first period of his watch (ie. the hours of the usual first phase of sleep), the constable has woken and is being watchful; either because he is feeling physically ill or, in compensation, lest he fall asleep again (‘sick’ perhaps being used in the sense of ‘guilty’ as well as indicating physical infirmity).^50^ Davenant might even be referring to a constable new to his duties who has slept for the first time on ‘a cold bench’, that is to say, has had the first taste of the harsh physical circumstances of his job. While it is impossible to determine the correct meaning of what might have been either a new coinage by Davenant or a colloquial phrase, it does lend itself to more than one interpretation.

Besides the night watch of Davenant’s constable, other sources cited by Ekirch involve unusual sleeping situations and arrangements. As Ekirch notes, William Baldwin’s *Beware the Cat* contains a reference to a ‘first slepe’; however, this is in the context of a Christmas gathering at Court, where the narrator and three other men share a chamber. Outside of the festive season and these sleeping arrangements—described at some length, suggesting novelty—an intellectual dispute in the middle of the night following a ‘first slepe’ may not have been normal behaviour:

It chaunced that at Christmas last I was at the Court with master Ferres then master of the Kyngs maiesties pastimes about the setting forth of certaine enterludes, which for the kynges recreation we had deuised and were in learning. In which time among many other exercises among our selues we vsed nightly at our lodging to talke of sundry thinges for the furtheraunce of such offices wherin ech man as than serued. For which purpose it pleased master Ferres to make me his bedfellowe, and vpon a pallet cast vpon the rushes in his owne chamber to lodge master willot and. M. Streamer, the one his Astronimor the other his diuine. And among many other thinges to long to reherse, it hapned on a night which I thinke was the. xxviij. of December after that M. Ferres was come from the court and in bead, there fell a controuersie betwene M. Streamer who with M. willot had already slept his first slepe, and I that was newly come to bead the effect wherof was whether birdes and beastes had reason […].^51^

While the phrase ‘first slepe’ is used here in a way that suggests a commonly understood phenomenon, it does not follow that this refers to a discrete period of sleep separated from a subsequent period by ‘up to an hour’ of wakefulness; this point is discussed in further detail below.

In the same vein as Davenant and Baldwin, the references to a first sleep in George Fidge’s *The English Gusman* do not describe normal circumstances for the middle or lower sorts. The text is a biography of the English highwayman James Hind; his ‘first sleep’ is taken ‘by the *Counter*’ (or compter), ie. a prison, while intoxicated.^52^

Hind being now come to *London*, did meet with many of his friends, and acquaintance, and one night being *drinking* in the *City*, and too long staying by the *good liquor*, made Indentures as he went by the *Counter* (a Trap to catch such *Rats*) was forced to take a nap before he went any further, and after his first *sleep*, awaked and looked about him, saying, *This is a large house and may entertain many guests*, but I do not intend to keep my Christmas here […].^53^

A later reference to a ‘first sleep’ in the same text appears to imply that waking in the night and being unable to return to sleep was unusual: ‘*Allen* [Hind’s fellow outlaw, fearing capture] made as though that disturbance would not let him sleep any more that night: saying, *When my first sleep is broke, I can sleep no longer*: so he sends one of his servants to the Host of the house to come and drink with him’.^54^ The fact that Allen feels the need to state ‘*When my first sleep is broke, I can sleep no longer*’ suggests that the alertness that follows—sending to the host to start a drinking session—is an exceptional rather than a routine practice. The phrase ‘*When my first sleep is broke*’ is itself ambiguous; it might mean that it is routine to have a break of consciousness following a ‘first sleep’, but unusual to then stay awake for the rest of the night, or that waking after a first period of sleep is not routine and may result in subsequent insomnia.

In other cases, the reference to a ‘first sleep’ is not accompanied by a description of a period of wakefulness between sleeps. For example, in Richard Hurst’s translation of Jean Ogier de Gombauld’s *Endimion*, it is not certain if the subject wakes up for ‘up to an hour’ during the night: ‘I tooke my first sleepe, which was short and quiet: but in my second I was much troubled with Dreames’.^55^ Indeed, even if we assume that there is ‘up to an hour’ interval of wakefulness, this entry is not consistent with Ekirch’s view that the period in between a first and second sleep was of special significance in meditating on and committing dreams to memory: here, the dreams of interest do not occur until the *later* part of sleep, although of course it is possible that this was an exception to a norm. In another of Ekirch’s references, in which ‘dead sleep’ is taken as a synonym for ‘first sleep’—James Shirley’s *The Constant Maid* (1640)—the text reads: ‘All people are a bed, the verie Owles/Are in their dead sleep’.^56^ This might simply be Shirley’s way of conveying the lateness of an hour at which no-one is up and about, not even night owls, rather than an indication that he believes owls have segmented sleep.

In brief, the phrase ‘first sleep’ is not of itself indicative of a habitual pattern of well-defined, separate periods of sleep, but might have other meanings depending on the context. Searching the Early English Books Online (EEBO) database for the term ‘first sleep’ in English printed texts with no date restrictions (thus covering the years 1475–1700) yields many such examples.^57^ ‘First sleep’ might mean an allocated period of sleep during shift activity, such as a military operation. The anonymous author of the English Civil War text *The Civil Wars of Bantam* (1683) describes sailors ‘dividing our selves into two Watches, my Lot being in the first. After Midnight, I went to Rest with somewhat better Hopes than those that had the first Sleep; as conceiving, if they had any real Design upon us, we should have heard of it before that time.’^58^ Alternatively, ‘first sleep’ might simply mean the initial period of sleep during a night of broken rest, consisting of interspersed sleep and waking. In *The speeches, discourses, and prayers, of Col. John Barkstead, Col. John Okey, and Mr. Miles Corbet*, politician and regicide Miles Corbet is quoted as stating, on the day of his execution, that ‘my first sleep […] from the time I went to bed, continued till two a clock, and I have had two sleeps since’.^59^

‘First sleep’ might also indicate the sleep preceding very transient, even incomplete, wakefulness: for example, in Richard Blackbourn’s novel *Clitie* (1688), the protagonist’s father experiences a significant dream during a second period of sleep after a moment of awakening: ‘after his first Sleep (which we’ll suppose was sound enough) he awak’d with a certain heaviness at his Heart, unlike to any he had felt before, but he continued not long ere soft slumbers clos’d his Eyes again’.^60^ Finally, ‘first sleep’ may simply refer to the belief that sleep is deepest in the early hours. Joseph Caryl’s *Exposition upon the Book of Job* (1656) interprets the reference to ‘that part of the night, *when deepe sleepe falleth on men*’ (Job 4:13) as meaning ‘in the former part or beginning of the night *for the first sleepe is the deepe sleepe;* and we use to say that a man, especially a weary hard-wrought man, is in a *dead sleepe,* when he is in his *first sleepe.*’^61^

It is perhaps unsurprising that ‘first sleep’ is a phrase with such diverse meanings. The OED’s definition of ‘first’ is notably capacious, indicating that the word might, in the early modern period as now, have meant ‘earliest in time or serial order, foremost in position, rank, or importance’.^62^ Among these possible meanings ‘first’ might simply indicate the initial phase of something; a lyric cited from 1500 uses the phrase ‘fyrste of wynter’ to describe the opening period of the season, rather than one winter that is followed by another: ‘The fyrste of wynter harde se shall ye … But the latter ende of wynter ys gude’. Similarly, when Philip Sidney wrote of Homer and the Greeks: ‘as by him their learned men tooke almost their first light of knowledge, so their actiue men, receiued their first motions of courage’, he was using the word ‘first’ to refer to the initiating (and valuable) stirrings of continuous processes, not implying that the ‘first light’ and ‘first motions’ were entities separated by a gap of time from subsequent lights of knowledge or motions of courage.^63^

The existence of the contrasting term ‘second sleep’ does not necessarily imply a gap between the two periods of sleep, or, if it does, it does not mean that first and second sleeps were clearly defined and separated periods in a pattern to which the majority of people rigidly adhered. Searching EEBO for the terms ‘second sleep/sleepe/slepe’ in full text with the same parameters used above retrieves fifteen hits.^64^ Of these, only five contain explicit references to both a ‘first’ and a ‘second’ sleep. In two cases, a ‘second sleep’ does not seem to mean a defined period of sleep at all. In his *sermon preached to the honourable Society of Lincolns-Inne* (1664), Ralph Cudworth writes:

And indeed if men should be restored after death to such gross, foul and cadaverous Bodies as these are here upon Earth, which is the very Region of Death and Mortality, without any change at all; what would this be else but, as Plotinus the Philosopher against the Gnosticks writes, […] to be raised up to a Second Sleep, or to be entombed again in living Sepulchres?^65^

A ‘Second Sleep’ is presented here as an undesirable absurdity; inhabiting a mortal body after death is like getting up only to fall asleep again. Similarly, in William Perkins’s *A godlie and learned exposition upon the whole epistle of Iude* (1606), the term ‘second sleep’ is used not to describe a literal segment or phase of sleep, but to indicate one of three types of ‘spirituall sleep’:

This spirituall sleep is three-fold: first, the naturall sleepe of heart by which euery one is ouertaken; so as by nature no man can so much as moue himselfe to the least good, till God awake him, and say to him, *Awake thou that sleepest, and stand vp from the dead.* The second sleepe is a slumber, and indeed the *remainders* of this naturall sleepe in the children of God, being awakened out of their dead sleepe; for euen they are ouertaken often with a spirituall slumber, by reason of remainders of sin in them. […] The third sleepe is the *increase of that naturall sleepe* and deadnes of heart by the custome of sinne, when as the heart is made past feeling, and altogether senselesse through continuance in sinne [.]^66^

When a ‘second sleep’ is something that follows on a ‘first sleep’, it is not always clear that this is a routine occurrence. For example, Moïse Amyraut’s *Discourse concerning the divine dreams mention’d in Scripture* (1676) tells the story of a man who dreams of his friend’s murder:

After Supper, he in the private house being gone to bed, and asleep, the other appear’d to him in a dream, and prayed him to come to his assistance, for as much as the Master of the Inn design’d to murther him: the affright of the dream having wakened him, he rose up, but being come to himself he took it for a meer dream and idle vision, and went to sleep again. In his second sleep, the image of his friend came again into his phansie [.]^67^

Arguably, the man in this tale wakes not because of a routine break in sleep, but because of a terrifying dream; he rises because of ‘affright’, but falls asleep again as soon as he is ‘come to himself’.

Even the time at which a ‘first sleep’ supposedly ends is not entirely consistent across early modern texts. Kenelm Digby’s *Choice and experimented receipts* (1675) advises: ‘Eat of these the quantity of three or four Dates in a morning after your first sleep, and sleep an hour or two upon it before you rise’, suggesting that ‘first sleep’ ends in the early hours of daylight, not midnight.^68^

While the possibility of segmented sleep cannot be ruled out by any of these texts, they demonstrate that the meaning of the phrases ‘first sleep’ and ‘second sleep’ are not fixed. In short, the extensive references containing the term ‘first sleep’ do not of themselves provide a weight of evidence for segmented sleep as a typically understood, widespread practice in early modern Europe. There are similar problems with the use of phrases such as ‘second sleep’ and ‘morning sleep’.

Similar issues may also arise when considering some of the evidence that Ekirch presents in other work from later periods. For example, in his 2015 paper published in *Past and Present*, ‘The Modernization of Western Sleep’, Ekirch writes that

In the early modern era, first and second sleep had generally been of equal duration, approximately three to three and a half hours apiece. But a distinct imbalance began to emerge by the mid-1800s, marked by a gradual expansion of the first phase to five or six hours at the expense of the second. […] Toward the end of the century, if not earlier, second sleep often did not commence until dawn or later, depending upon the season. ‘How long does our “first sleep” last?’ asked a British paper in 1891. According to the renowned psychiatrist Sir James Crichton Browne, the ideal was eight hours for ‘actively working brains’.^69^

However, a fuller quotation from the cited text gives a slightly different picture. It reads:

How long does our ‘first sleep’ last? Sir James Crichton Browne […] told his hearers that ordinary sleep grows deeper for the first hour and a half, and then steadily diminishes until the slumberer awakens. Dr. Browne pleads for eight hours for actively working brains [.]^70^

It is therefore unclear whether Crichton-Browne is referring to the first phase of sleep of 90 minutes or the consolidated block of sleep of 8 hours as the ‘first sleep’. The reading that Crichton-Browne is using ‘first sleep’ to refer to a relatively short but beneficial first phase of sleep, as opposed to referring to an eight-hour ‘first sleep’ is reinforced by a later essay, in which he writes:

It is but reasonable to suppose that the recuperative power of sleep is proportionate to its intensity, and it appears therefore that experiment confirms that popular belief that **the ‘first sweet sleep of night,’ or the ‘beauty sleep,’ as it is popularly called, is the best, and that one hour’s sleep before midnight is more restorative than two hours after it.** It is probable that the curve of intensity of sleep varies somewhat with the individual and with surrounding circumstances, but it may be laid down as a general rule that it is in the first part of a night’s sleep that the products of fatigue are most rapidly removed from the brain and that repairs are most promptly executed, and in the case of children the ground cleared for growth. It follows that it is that part of the night’s sleep that should be most jealously guarded and kept tranquil and free from sensory disturbances, and that it is peculiarly important that it should be secured unbroken to children. [My emphasis]^71^

In other words, ‘first sleep’ is being used here to describe a first phase of sleep of an hour or two before midnight that is thought to be particularly beneficial, with no evidence that this is necessarily followed by a period of wakefulness. It is doubtful that Crichton-Browne is referring in his 1891 lecture to an eight-hour extended ‘first sleep’. Terms such as ‘first sleep’, ‘dead sleep’, ‘second sleep’ and ‘morning sleep’ should therefore invite cautious interpretation whenever they are found, whether in pre- or post-industrial texts.

## Nocturnal activity in early modern England

As noted above, broken sleep was experienced commonly in the early modern period, as it is today. The question remains as to whether the textual evidence cited by Ekirch suggests a general pattern of a period of ‘up to an hour’ of wakefulness, as opposed to a more diverse picture of transitory waking, or waking because of specific professional or personal pressures (for example, to do housework or for religious reasons). For example, while Ekirch quotes a line from George Wither’s *Iuvenilia* (1633) – ‘At mid-night when thou wak’st from sleepe’ – to demonstrate that wakefulness at night was normalised, putting this excerpt in context reveals a more complex picture. The poem concerns ‘Presumption’, and Wither is attempting to stir the subject’s guilty conscience, emphasising the terror and isolation of the night:

Reader, if this do no impression leaue,
So that thou canst not any feare conceiue
Through this description; thinke vpont at night
Soone in thy bed when earth’s depriu’d of light
I say at mid-night when thou wak’st from sleepe,
And lonely darknesse doth in silence keep
The Grim-fac’t night.^72^

All that can be gleaned from this text is that the sleep of the reader might be broken, whether from external causes such as noise, internal feelings of guilt or for no particular reason. It is true that one interpretation of this text is consistent with Ekirch’s idea of first and second sleeps; however, at the risk of repetition, other readings are possible, and incidences of unwanted broken sleep cannot always be clearly distinguished from routinely segmented sleep.

Similarly, Ekirch writes that ‘in the view of John Locke, “That all men sleep by intervals” was a common feature of life’; however, other readings are possible when the quotation is placed in context.^73^ The relevant section of Locke’s *An Essay Concerning Humane Understanding* (1690) concerns the impossibility of pronouncing absolute knowledge on man:

[W]e cannot with Certainty affirm, That all Men sleep by intervals; That no Man can be nourished by Wood or Stones; That all Men will be poisoned by Hemlock: because these Ideas have no connexion nor repugnancy with this our nominal Essence of Man, with this abstract Idea that Name stands for.^74^

The passage in question uses human sleep patterns in passing as an example, rather than specifically focusing on this topic. While one reading of it is congruent with Ekirch’s argument—sleeping ‘by intervals’ is, like the absence of wood- and stone-based diets, a self-evident, if ultimately unprovable truth—Locke is vague regarding what sort of interval he means and how long such intervals last. He could mean that segmented sleep is universal; he could mean that sleep is often subject to transient interruptions; or his meaning could be that people alternate between wakefulness during the day and unconsciousness at night. Elsewhere, Locke refers to individuals ‘who sleep out whole stormy Nights, without hearing the Thunder’, which is not suggestive of segmented sleep as the norm.^75^ In brief, multiple meanings were attached to the terminology around sleep, resulting in confusion for the modern-day reader about what constituted ‘normal’ sleep (or even if such norms existed). Ambiguous evidence which might refer to segmented sleep, or simply something very like broken sleep as we would recognise it today, is not made clearer or stronger by its accretion.

Ekirch’s description, as noted above, paints a picture of the early modern night as a time of activity and even sociability, an opportunity afforded by segmented sleep: ‘Families rose from their beds to urinate, smoke tobacco, and even visit close neighbors. Remaining abed, many persons also made love, prayed, and, most important, reflected on the dreams that typically preceded waking from their “first sleep”.’^76^ But it is not clear that these activities were either common in practice or communal in nature. For example, urination in the night is indeed mentioned in medical texts such as Andrew Boorde’s *Compendyous Regyment* (1547); however, getting out of bed to urinate is not an activity unique to pre-industrial societies, and the passage as a whole suggests that the individual will alternate between sleep and brief periods of waking, rather than experiencing two distinct segments of sleep:

whan you be in your bed lye a lytel whyle on your left syde, & slepe on your ryght syde. And whan you do wake of your fyrste slepe make water yf you fele your bladder charged, and then slepe on the lefte syde, and loke as ofte as you do wake so ofte tourne your selfe in the bed from the one syde to the other.^77^

In other words, the passage could suggest that people in the early modern period might have expected to wake periodically; the ending of the first period of sleep may have been one such occasion (and been accompanied by the need to urinate), and the phrase ‘as ofte as you do wake’ suggests that there may have been other brief moments of consciousness during the night. Boorde’s advice was to use such occasions to turn and sleep on the other side. One possibility is that Boorde is distinguishing between a ‘up to an hour’ of wakefulness following a ‘fyrste slepe’ that should be used to discharge the bladder, and later, briefer moments used simply to turn in bed. It is also possible that ‘as ofte as you do wake’ refers to the moment of waking after a first sleep across a series of nights, rather than multiple incidences of waking within the same night. It may, however, also be a passage that reflects the idiosyncrasies of individual sleep patterns, rather than providing evidence for a standardised time for nocturnal urination, as ‘families’ or otherwise.

Ekirch’s other examples of nocturnal activity again suggest a diversity of patterns of sleep and activity based on individual circumstances, rather than a common period of ‘up to an hour’ of wakefulness.^78^ The legal depositions that Ekirch cites refer to individuals rising from bed for a specific purpose—for example, studying, checking for marauding cows, stealing things, or leaving the house with other illicit or criminal plans—and do not demonstrate that these episodes of activity were part of a normal night’s sleep pattern. Furthermore, the statement that ‘women left their beds to perform myriad chores’ elides ‘women’ with ‘servants’: one example cited is the deposition of a servant rising to brew malt, and the other a protest poem by manual labourer Mary Collier.^79^ In other words, these are examples of nocturnal activity made necessary by an individual’s social and professional position, rather than spontaneous activity during a natural break in sleep.

There is, of course, the question as to how people in the early modern period would have woken up without the aid of alarm clocks. Sasha Handley has suggested factors such as the orientation of beds facing east to catch the sun’s rays, and external noises (for example, from animals) may helped to wake people in the morning. Although these could have ensured early rising in the summer months, it is less likely that they would have worked in the dark winter mornings. Another factor might simply have been a mixture of motivation and habituation; Handley suggests that this might have aided spiritually influenced early rising. This topic requires further research; however, for the purposes of the present article, it is sufficient to note that domestic staff of the early modern period were obliged to rise in the night and did so, and that this fact does not of itself present evidence for segmented sleep. The degree of obligation and compulsion experienced by servants means that it is hard to ascertain how spontaneous their early morning waking might have been.^80^

While Ekirch writes—qualifying his earlier statement ‘Families rose from their beds’—that ‘Most people, upon awakening, probably never left their beds unless to relieve their bladders, if then’, he also suggests that the early modern night might have been at least somewhat sociable. ‘Besides praying’, he writes, the people of the early modern period ‘conversed with a bedfellow or inquired about the well-being of a child or spouse.’^81^ Depositions and diaries support the existence of nocturnal conversations: however, these confirm that people did indeed wake up in the night, but not necessarily that this was part of a common pattern of ‘first’ and ‘second’ sleep separated by a period of ‘up to an hour’.

Ekirch also points to a print by Dutch artist Jan Saenredram, *Night* (c. 1595), which shows nocturnal activity, in this case a wife adjusting her husband’s bedding.^82^ But as with the textual evidence, this picture also provides evidence for a counter-argument. First, the wife is the only person awake in the room, while her husband and maid sleep: thus, interrupted sleep is not depicted as a social phenomenon, accompanied by nocturnal conversation or inquiry. All that we as twenty-first-century viewers can tell from the picture is that the wife has woken up, with no indication as to how long this period of wakefulness will last or what behavioural norms attend such an event. Second, the inscription at the foot of the picture indicates that the purpose of the picture is to depict night as a time of peace, not as a moment of activity between sleeps: ‘Nocte vacant curis animi, placidamq*ue* quietem/ Percipiunt, gratoq*ue* indulgent omnia somno’; ‘At night minds are free from cares, they perceive placidity and quiet, and they indulge all things in welcome sleep.’^83^ This is a vignette of a period of solitary, perhaps brief, wakefulness, against a general backdrop of oblivion. Even if the inscription has an ironic flavour, given the depiction of a wife attending to her sleeping husband’s comfort, this nevertheless reinforces the ideal of the night as a time of ‘placidity and quiet’. Furthermore, the height of the fire in the grate—as opposed to burned-down wood or coals—casts doubt on what time precisely is represented here. While it would be unlikely that a single image could make a conclusive case for segmented sleep, it is unclear in what way this specific picture supports the theory of ‘first’ and ‘second’ sleeps separated by ‘up to an hour’.

## Conclusion

The model of segmented pre-modern sleep proposed by Roger Ekirch two decades ago has been widely accepted by historians, literary scholars and the general public. However, the scientific and anthropological evidence presented in support of this model is selective and limited. While the evidence from early modern texts is impressive in its breadth, close analysis reveals a significant degree of ambiguity and nuance that argues against a routinely segmented sleeping pattern as the sole or even the main meaning attributable to references to the ‘first sleep’, ‘dead sleep’ or ‘second sleep’. The ‘first’ and ‘second’ sleeps might simply represent different phases of a continuous process of sleep; if there was a gap, it might have been so short as to be insignificant in terms of offering opportunities for nocturnal activity; nocturnal activity in any case might have related more to the specific circumstances of the individual (for example, domestic service) rather than being a generalised, society-wide phenomenon. Some texts might simply be describing disturbed sleep – a feature not unique to the early modern world – and, in some cases, the inability to return to sleep after waking, which would today be classified as middle or terminal insomnia. Evidence from printed sources, meanwhile, indicates that the terms ‘first sleep’ and ‘second sleep’ held more than one meaning, and that each individual use of the term needs contextualisation both within the early modern text itself and preferably with collateral information regarding the author and circumstances of the specific text’s production.

The present reconsideration of the segmented sleep theory has limitations. The sheer volume of literary sources cited by Ekirch would make re-analysis of every citation prohibitively long. However, the examples quoted here illustrate that reading these sources without the assumption of segmented sleep as the early modern norm reveals alternative meanings. Moreover, to reiterate a point made above, ambiguous sources are not made any less ambiguous through collation. Constructing models of the early modern night is, by nature, a tentative, limited and ambiguous process; the readings which this article proposes are undoubtedly open to further revision.

There are two main implications of this research. First, there is a greater diversity of interpretation of sleep-related texts than might be assumed from the widely accepted segmented sleep model. Segmented sleep may have been practised by some individuals; however, it was not the sole or even necessarily the predominant model of sleep in the early modern period. Second, literary critics and cultural historians should look beyond the assumption that segmented sleep was routine when examining important aspects of nocturnal early modern culture, such as the experience of dreaming. Ekirch’s work has proved hugely valuable to the fields of early modern cultural history, the history of medicine and modern-day sleep studies in both collating a huge amount of material and questioning basic assumptions about human sleeping patterns. The intention of the present article is to prompt new, detailed and complex readings of this and other work related to early modern sleeping and dreaming.

## Footnotes

1

Roger A. Ekirch , ‘Sleep We Have Lost: Pre-industrial Slumber in the British Isles’, American Historical Review, 106 (2001), 343–386 (p. 344)18680884.

2

Craig Koslofsky , Evening’s Empire: A History of the Night in Early Modern Europe (Cambridge: Cambridge University Press, 2011), p. 6 .

3

Claude Fretz , Dreams, Sleep, and Shakespeare’s Genres (Cham: Palgrave Macmillan, 2020), 52 .

4

Tara Hamling and Catherine Richardson , A Day at Home in Early Modern England: Material Culture and Domestic Life, 1500–1700 (New Haven and London: Yale University Press, 2017), 260 .

5
*A Family Guide to ‘To Sleep, Perchance to Dream’*, Folger Shakespeare Library, 2009 <https://folgerpedia. folger.edu/mediawiki/media/images_pedia_folgerpedia_mw/f/f1/To_Sleep_Perchance_to_Dream_FAMILY_GUIDE.pdf> [Accessed 10 April 2022].

6

Brian Fagan and Nadia Durrani , What We Did in Bed: A Horizontal History (New Haven andLondon: Yale University Press, 2019), 5 .

7 Stephanie Hegarty, *The myth of the eight-hour sleep*, online news report, BBC News, 22 February 2012 <https://www.bbc.co.uk/news/magazine-16964783> [Accessed 2 February 2022].

8 Katie Gatens, ‘Wake up to the wonders of a broken night’s sleep’, *The Sunday Times*, 20 February 2022, <https://www.thetimes.co.uk/article/wake-up-to-the-wonders-of-a-broken-nights-sleep-hg8c9hzp7> [Accessed 8 May 2022].

9

Robert Harris , The Second Sleep (London: Hutchinson, 2019), vii, 19 .

10 Three references (to Noel Taillepied’s work, a letter by Erasmus and a lecture by James Crichton-Browne) were added following comments arising from the peer review process; I am grateful to reviewers for drawing my attention to these texts.

11 Ekirch, ‘The Modernization of Western Sleep: Or, Does Insomnia Have a History?’, *Past and Present*, 226 (2015), 149–192 (154). He adds that ‘they are also well represented in medieval literature and, though fewer in number, in […] classical works’.

12 See A. Roger Ekirch , At Day’s Close: A History of Nighttime (London: Weidenfeld & Nicolson, 2005); Ekirch, op. cit. (note 11); A. Roger Ekirch . ‘Segmented *Sleep* in Preindustrial Societies’, Sleep, 39 (2016), 715–716 2688845410.5665/sleep.5558PMC4763365; A. Roger Ekirch , ‘What sleep research can learn from history’, Sleep Health, 4 (2018), 515–518 3044231910.1016/j.sleh.2018.10.004. Numerous excerpts from primary sources are also collected on Ekirch’s website, *Additional Historical References to “Segmented Sleep”* <https://sites.google.com/vt.edu/roger-ekirch/sleep-research/segmented-sleep> [Accessed 3 September 2022]. In terms of the 2001 article’s influence, it is cited in the work by Fretz [*op. cit.* (note 3)] and Koslofsky [*op. cit.* (note 2)] mentioned at the start of this article (note that Koslofsky and Fretz also cite *At Day’s Close*, which draws on many of the same sources as the 2001 article in its sections on segmented sleep and, indeed, cites this article. Hamling and Richardson (*op. cit.* (note 4)] cite *At Day’s Close*, as do Fagan and Durrani [*op. cit.* (note 6)].

13 In the case of ‘The Modernization of Western Sleep’, Ekirch [*op. cit. (* note 11)] reviews material from the late eighteenth century to the early twentieth century; an example from this article is discussed below.

14

Janine Rivière , Dreams in Early Modern England (New York and Abingdon: Taylor and Francis, 2017), 146 .

15 Ekirch, *op. cit.* (note 1), 364.

16

Sasha Handley , Sleep in Early Modern England (New Haven: Yale University Press, 2016), 8–9. Handley cites Ekirch’s ‘Sleep We Have Lost’, *op. cit.* (note 1), *At Day’s Close*, *op. cit.* (note 12) and ‘Modernization’, *op. cit.* (note 11).

17 Gabriele Schichta, private correspondence, 23 November, 2020, quoted with permission.

18 Handley, *op. cit.* (note 16), 213.

19 This phrasing was modified by Ekirch in 2015, when he stated that the interval between sleeps was ‘Typically […] bridged shortly past midnight by up to an hour or so of wakefulness’. op. cit. (note 13), 152.

20 Ekirch, op. cit. (note 1), 144.

21

Patrick McNamara , The Neuroscience of Sleep and Dreams (Cambridge: Cambridge University Press, 2019), 30–32 .

22 Rivière, op. cit. (note 14), i68–i69 (i68).

23

Alexander Marr , ‘Richard Haydocke’s *Oneirologia*: A Manuscript Treatise on Sleep and Dreams, including the “Arguments” of King James I’, Erudition and the Republic of Letters, 2 (2016), 113–81 (p. 171).

24 Ekirch, *op. cit.* (note 1), 381.

25 MS Ashmole 1478, Bodleian Library, Oxford, UK, fol. 48^v^.

26 Fretz, for example, notes Ekirch’s argument that ‘dreams occurring during the “first sleep” would have been “unusually vivid, and their images would have intruded far less on conscious thought had sleepers not stirred until dawn”’ and adds that ‘If Ekirch is right, then the early modern physician and clergyman Timothy Bright’s observations that dreams are indistinct from waking reality, and that they can make the past and the future seem like the present are more literal descriptions and refer to more powerful phenomena than our modern experiences of dreaming might lead us to believe’. Fretz, *op. cit.* (note 3), 52.

27 For clarity, Ekirch himself does not propose that ‘natural’ practices are of themselves healthier than ‘unnatural’ ones.

28 Ekirch, *op. cit.* (note 1), 367–368.

29

Thomas A. Wehr , Douglas E. Moul , Giuseppe Barbato , Holly A. Giesen , Jason A. Seidel , Charles Barker , Charles Bender , ‘Conservation of photoperiod-responsive mechanisms in humans’, American Journal of Physiology (1993), 265, R846–R857 (R846)823845610.1152/ajpregu.1993.265.4.R846.

30
*Ibid.*, R846–R847.

31 Natalie Angier, ‘Modern Life Suppresses Ancient Body Rhythm’, *New York Times*, 14 March 1995 <https://www.nytimes.com/1995/03/14/science/modern-life-suppresses-an-ancient-body-rhythm.html> [Accessed 23 June 2019].

32 It should also be noted – in the interests of complete reporting – that one participant ‘progressively became severely depressed and suicidal during the first 5 days of exposure to long nights and was removed from the experiment.’ Wehr, *op. cit.* (note 29), R847, R848, R852.

33

Kenneth P. Wright Jr , Andrew W. McHill , Brian R. Birks , Brandon R. Griffin , Thomas Rusterholz , Evan D. Chinoy , ‘Entrainment of the Human Circadian Clock to the Natural Light–Dark Cycle’, Current Biology, 23 (2013), 1554–1558 2391065610.1016/j.cub.2013.06.039PMC4020279.

34 Ekirch, *op. cit.* (note 1), 367.

35

Paul Bohannan , ‘Concepts of Time among the Tiv of Nigeria’, Southwestern Journal of Anthropology, 9 (1953), 251–262 (253).

36 Rivière, *op. cit.* (note 14), 137.

37 Hamling and Richardson, *op. cit.* (note 4), 260.

38

Thomas Bentley , The monument of matrones (London: Henry Denham, 1582), sig. Dddd2^r^.

39 Ekirch, *op. cit.* (note 1), 366. Noel Taillepied , A Treatise of Ghosts, trans. Montague Summers (London: The Fortune Press, 1933), 97–98 .

40 Taillepied, *ibid.* 97–98. Noel Taillepied , Psichologie ou Traité de l’Apparition des Esprits (Paris: Guillaume Bichon, 1588), sig. G13^v^: ‘enuiron la minuict quand on est eueillé du premier somme, lors que les sens sont libres & en repos’.

41 Taillepied, transl. Summers (note 39), 87.

42
*Additional Historical References to “Segmented Sleep”* < https://sites.google.com/vt.edu/roger-ekirch/sleepresearch/segmented-sleep> [Accessed 3 September 2022].

43 James M. Estes, ‘Erasmus’ Illnesses in His Final Years (1533–1536)’ in James M. Estes (ed.), Desiderius Erasmus, *The Correspondence of Erasmus*, trans. Clarence Miller (Toronto: University of Toronto Press, 2020), 336–338.

44
*Op. cit.* (note 16), 74.

45 Lewis Bayly, *The New Practice of Piety* (London: 1749), sig. G6^r^.

46 ‘ejaculation, *n.*’, *OED Online*, Oxford University Press, June 2022 <www.oed.com/view/Entry/60031> [Accessed 4 September 2022].

47

Maurice M. Ohayon , ‘Nocturnal Awakenings and Difficulty Resuming Sleep: Their Burden in the European General Population’, Journal of Psychosomatic Research, 69 (2010), 565–571 2110904410.1016/j.jpsychores.2010.03.010.

48 Ekirch, *op. cit.* (note 1), 364. Footnote states he has ‘discovered sixty-three references within a total of fifty-eight different sources from the period 1300–1800’.

49 Ekirch, op. cit. (note 1), 366; William Davenant, *The Unfortunate Lovers* (London: 1643), sig. A4^v^; sig. G1^v^.

50 ‘sick, *adj.* and *n.*’, *OED Online*, Oxford University Press, December 2021 <www.oed.com/view/Entry/179187> [Accessed 26 February 2022].

51 William Baldwin, *Beware the Cat* (London: 1570), sig. A3^r^.

52 ‘compter, *n.*’, *OED Online*, Oxford University Press, December 2021 <www.oed.com/view/Entry/37923> [Accessed 26 February 2022].

53 George Fidge *The English Gusman* (London, 1652), sig. B3^r^.

54
*Ibid.*, C2^v^–C3^r^.

55 Jean Ogier de Gombauld, *Endimion*, trans. Richard Hurst (London: 1639), sig. F5^v^.

56 Ekirch, *op. cit.* (note 1), p. 365: ‘For references to the “first sleep” of animals, see, for example, James Shirley, *The Constant Maid* (London, 1640)’. James Shirley, *The Constant Maid* (London: 1640), sig. F1^r^.

57 EEBO is an online resource which ‘aims to provide digital images of one copy each of the surviving books and broadsides printed in the British Isles and British North America between 1473 and 1700 and of the English-language books printed in Europe during the same period.’ EEBO full-text searches are limited by their restriction to printed texts, the survival of such texts, their transcription via the Text Creation Partnership (not all books on EEBO have been transcribed) and the accuracy of these transcriptions and indexing. Joseph Loewenstein and Alireza Taheri Araghi, *EEBO and EEBO-TCP: A Brief Introduction* <https://earlyprint.org/intros/intro-to-eebo-and-eebo-tcp.html> [Accessed 9 October 2022].

58
*The Civil Wars of Bantam* (London: 1683), sig. B2^r^.

59 John Barkstead, *The Speeches, Discourses, and Prayers, of Col. John Barkstead, Col. John Okey, and Mr. Miles Corbet* (No location: 1662), sig. E4^v^.

60 Richard Blackbourn, *Clitie* (London: 1688), sig. K2^r^.

61 Joseph Caryl, *An Exposition upon the Book of Job* (London: 1656), sig. M4^r^.

62 ‘first, *adj.*, *adv.*, and *n.*2’, *OED Online*, Oxford University Press, March 2022 <www.oed.com/view/Entry/70609> [Accessed 10 April 2022].

63 Philip Sidney, *Defence of Poesie* (London: 1595), sigs. G3^r^–G3^v^.

64 The numerous limitations of EEBO searches are outlined in a previous footnote. Nevertheless, assuming that errors and omissions occur more or less at random (in that they are no more likely to affect the frequency of detection of one term than another), they can give a very rough indication of how common particular terms may have been in early modern print.

65 Ralph Cudworth, *A sermon preached to the honourable Society of Lincolns-Inne* (London: 1664), sigs. H3^r^–H3^v^.

66 William Perkins, *A godlie and learned exposition upon the whole epistle of Iude* (London: 1606), sig. G6^r^.

67 Moïse Amyraut, *A discourse concerning the divine dreams mention’d in Scripture*, trans. James Lowde (London: 1676), sig. C6^r^.

68 Kenelm Digby, *Choice and experimented receipts* (London: 1675), sig. G7^v^.

69 Ekirch, *op. cit.* (note 11), 15.

70 ‘This Morning’s News’, *Daily News (London)*, 6 March 1891.

71

James Crichton-Browne , Stray Leaves from a Physician’s Portfolio (London: Hodder and Stoughton, 1927), 127 .

72 George Wither, *Iuvenilia* (London: 1633), sig. M12^r^.

73 Ekirch, *op. cit.* (note 1), 365; (note 11), 154.

74 John Locke, *An essay concerning humane understanding* (London: 1690), sig. Qq1^v^.

75
*Ibid.*, sig. P4^v^.

76 Ekirch, *op. cit.* (note 1), 344.

77 Andrew Boorde, *Compendyous Regyment* (London: 1547), sig. C1^v^.

78 The word ‘common’ is used here following Ekirch’s own use of the term in ‘Sleep We Have Lost’ (note 1), 365: ‘in the view of John Locke, “That all men sleep by intervals” was a common feature of life’.

79 Ekirch, *op. cit.* (note 1), 370.

80 For Handley’s comments, see Emma Bryce, ‘How Did People Wake Up Before Alarm Clocks?’, *Live Science*, 3 November 2018. <https://www.livescience.com/64002-how-wake-up-before-alarm-clocks.html> [Accessed 5 September 2022].

81 Ekirch, *op. cit.* (note 1), 371.

82 Ekirch, *op. cit.* (note 1), 371–372. A high-resolution image is available at <https://www.nga.gov/collection/art-object-page.154310.html> [Accessed 8 May 2022].

83 My thanks to Lily Hart for assistance with this translation.

